# The Role of the Pancreatic Extracellular Matrix as a Tissue Engineering Support for the Bioartificial Pancreas

**DOI:** 10.3390/biomimetics9100598

**Published:** 2024-10-02

**Authors:** Thamires Santos da Silva, Leandro Norberto da Silva-Júnior, Bianca de Oliveira Horvath-Pereira, Maria Carolina Miglino Valbão, Matheus Henrique Herminio Garcia, Juliana Barbosa Lopes, Carlos Henrique Bertoni Reis, Rodrigo da Silva Nunes Barreto, Daniela Vieira Buchaim, Rogerio Leone Buchaim, Maria Angelica Miglino

**Affiliations:** 1 Graduate Program in Anatomy of Domestic and Wild Animals, Faculty of Veterinary Medicine and Animal Science, University of São Paulo (FMVZ/USP), São Paulo 05508-270, Brazil; thamiresssilva@usp.br (T.S.d.S.); silvajunior@usp.br (L.N.d.S.-J.); horvath@usp.br (B.d.O.H.-P.); rodrigobarreto@usp.br (R.d.S.N.B.); danibuchaim@alumni.usp.br (D.V.B.); rogerio@fob.usp.br (R.L.B.); 2 Postgraduate Department, University of Marília (UNIMAR), Marília 17525-902, Brazil; mariacarolmv@gmail.com (M.C.M.V.); matheushenrigarcia@gmail.com (M.H.H.G.); jublopes89@gmail.com (J.B.L.); 3 Postgraduate Program in Structural and Functional Interactions in Rehabilitation, University of Marilia (UNIMAR), Marília 17525-902, Brazil; dr.carloshenriquereis@usp.br; 4 UNIMAR Beneficent Hospital (HBU), Medical School, University of Marilia (UNIMAR), Marilia 17525-160, Brazil; 5 Department of Animal Morphology and Physiology, Faculty of Agricultural and Veterinary Sciences, São Paulo State University, Jaboticabal 14884-900, Brazil; 6 Medical School, University Center of Adamantina (UNIFAI), Adamantina 17800-000, Brazil; 7 Department of Biological Sciences, Bauru School of Dentistry (FOB/USP), University of Sao Paulo, Bauru 17012-901, Brazil; 8 Postgraduate Program in Animal Health, Production and Environment, University of Marilia (UNIMAR), Marilia 17525-902, Brazil

**Keywords:** extracellular matrix, insulin-producing cells, diabetes mellitus, tissue engineering, decellularization

## Abstract

Type 1 diabetes mellitus (T1DM) is a chronic condition primarily managed with insulin replacement, leading to significant treatment costs. Complications include vasculopathy, cardiovascular diseases, nephropathy, neuropathy, and reticulopathy. Pancreatic islet transplantation is an option but its success does not depend solely on adequate vascularization. The main limitations to clinical islet transplantation are the scarcity of human pancreas, the need for immunosuppression, and the inadequacy of the islet isolation process. Despite extensive research, T1DM remains a major global health issue. In 2015, diabetes affected approximately 415 million people, with projected expenditures of USD 1.7 trillion by 2030. Pancreas transplantation faces challenges due to limited organ availability and complex vascularization. T1DM is caused by the autoimmune destruction of insulin-producing pancreatic cells. Advances in biomaterials, particularly the extracellular matrix (ECM), show promise in tissue reconstruction and transplantation, offering structural and regulatory functions critical for cell migration, differentiation, and adhesion. Tissue engineering aims to create bioartificial pancreases integrating insulin-producing cells and suitable frameworks. This involves decellularization and recellularization techniques to develop biological scaffolds. The challenges include replicating the pancreas’s intricate architecture and maintaining cell viability and functionality. Emerging technologies, such as 3D printing and advanced biomaterials, have shown potential in constructing bioartificial organs. ECM components, including collagens and glycoproteins, play essential roles in cell adhesion, migration, and differentiation. Clinical applications focus on developing functional scaffolds for transplantation, with ongoing research addressing immunological responses and long-term efficacy. Pancreatic bioengineering represents a promising avenue for T1DM treatment, requiring further research to ensure successful implementation.

## 1. Introduction

**Figure 1:**
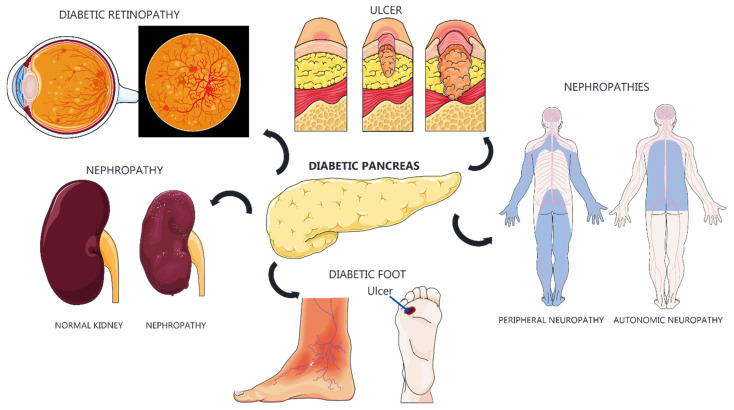
Diabetic complications and their systemic impacts. Diabetes can lead to severe complications affecting multiple organs and systems. Diabetic retinopathy compromises the retinal blood vessels, potentially leading to vision loss. Diabetic nephropathy causes progressive kidney damage, resulting in renal failure. Diabetic ulcers, particularly in the feet, occur due to poor circulation and neuropathy, which can result in amputations. The diabetic pancreas has impaired insulin production. Diabetic neuropathies include peripheral neuropathy, causing pain and loss of sensation in the limbs, and autonomic neuropathy, which affects involuntary functions such as digestion and blood pressure regulation. These complications underscore the importance of maintaining good glycemic control to prevent further systemic damage.

Type 1 diabetes mellitus (T1DM) is a chronic disease with significant treatment costs primarily attributed to the requirement for insulin replacement. T1DM is associated with complications such as vasculopathy [1], cardiovascular diseases [2,3], nephropathy [4], neuropathy [5], ulcers, and retinopathy (Figure 1).

In severe cases, pancreatic islet transplantation is an option, but its success does not depend solely on adequate vascularization [6,7]. The main limitations to clinical islet transplantation are the scarcity of human pancreas, the need for immunosuppression, and the inadequacy of the islet isolation process [8]. Despite numerous studies, diabetes mellitus remains a significant global health issue. Current treatment involves insulin administration, which manages symptoms but does not provide a cure, often leading to severe kidney and heart complications [9].

In 2015, diabetes affected approximately 415 million people worldwide [10], and it is projected to be one of the leading causes of death by 2030 [11,12]. Predictions suggest expenditures of USD 1.7 trillion on diabetes-related costs between 2011 and 2030 [12]. Pancreas transplantation is a potential option for curing diabetes, but its efficacy is limited by organ donor availability. The complexity of pancreatic vascularization is also a major obstacle because of its drainage pattern formed by multiple blood vessels [13].

T1DM is caused by the autoimmune destruction of insulin-producing pancreatic cells [3]. Biomedical advancements have led to technologies aimed at “encapsulating” beta cells and reducing autoimmune responses [14,15]. Biomaterial research has also advanced, offering new possibilities for tissue reconstruction and transplantation [16,17]. An important source of biomaterials is the extracellular matrix (ECM), which consists of a complex array of macromolecules such as proteins, proteoglycans, and glycosaminoglycans. These components perform structural and regulatory functions in cell migration, differentiation, proliferation, and adhesion [18,19,20,21]. Therefore, ECM has great potential as a biomaterial, not only providing tissue-structuring components but also maintaining the three-dimensional architecture required for transplanting and implanting biofunctional units in tissue reconstitution therapies [19].

Tissue engineering extends beyond tissue regeneration to encompass the manufacturing of new organs. In this context, the development of a bioartificial pancreas, which integrates insulin-producing cells, biologically active molecules for signaling cellular functions, and a suitable framework, could be an extremely promising alternative for treating T1DM [22].

Pancreas transplantation is rare in animals because of the high risk of rejection and complications such as pancreatitis and thrombosis [23]. Studies on diabetes, particularly type 2 (T2DM), have predominantly used rodents [24]. However, it is necessary to explore other animal models, such as pigs, dogs, and cats, which better mimic T2DM [25,26].

This article primarily focuses on the production of a decellularized pancreas followed by its recellularization. This approach was chosen because, based on a review of the literature, decellularization followed by recellularization appears to be the most promising method for creating an artificial pancreas [27,28,29,30,31]. However, the challenge in applying this method to diabetes lies in the selection of cells for transplantation. While pancreatic islets seem to be the most appropriate, transplantation remains problematic due to significant vascular barriers and complications associated with maintaining the organ’s exocrine function. Therefore, the aim of this work is to summarize the advances in pancreatic tissue engineering using decellularized ECM.

## 2. Fundamentals of Pancreatic Tissue Engineering

### 2.1. Anatomy, Physiology, and Endocrine/Exocrine Functions of the Pancreas

The pancreas is an extension of the glandular layer of the embryonic duodenum, remaining connected to this part of the intestine through exocrine secretory ducts. In certain species, the two primitive duodenal rudiments persist, whereas in others, one involutes, and only one excretory duct remains [32].

The excretory system of the pancreas varies among domestic species because of the regression of one or part of the primitive buds. In humans, horses and dogs, both the dorsal and ventral pancreatic buds persist. The main duct, stemming from the ventral bud, along with the bile duct, drains into the major duodenal papilla. The accessory duct, stemming from the dorsal bud, drains into the minor duodenal papilla [32,33]. In cattle and pigs, only the duct stemming from the dorsal bud (which would be mistakenly called the “accessory” pancreatic duct) persists, draining into the minor duodenal papilla. In cats and small ruminants, only the ventral bud remains, draining into the major duodenal papilla [32].

The pancreas exhibits structural similarities across species and undergoes rapid autolysis after death. Its exocrine portion secretes pancreatic juice containing enzymes for protein, fat, and carbohydrate digestion. The endocrine portion produces insulin and glucagon, and the organ excretory system, which is supplied by capillaries, carries these hormones through the blood system via diffusion [32,34].

The pancreas is intricately vascularized, typically decellularized for research purposes using perfusion with detergents injected via various routes [35]. The pancreas is irrigated by numerous arteries originating from either the celiac artery or the mesenteric celiac trunk, depending on the species. These arteries include the right gastric artery, gastroduodenal artery, cranial pancreaticoduodenal artery, cranial mesenteric artery, and caudal pancreaticoduodenal artery. Venous drainage occurs through tributaries of the portal vein, with contributions from the gastroduodenal vein and caudal mesenteric vein forming the portal vein [32].

The complex vascularization and excretory system of the pancreas highlight the necessity for alternative decellularization methods and subsequent endocrine recellularization strategies. Efforts to find vascularized sites for pancreatic transplantation must consider insulin production and its transportation through the circulatory system [32].

### 2.2. Challenges Related to Manufacturing Bioartificial Pancreases, Including the Precise Reproduction of Cellular Architecture and the Maintenance of Secretory Function

The pancreas has a highly complex and organized structure, characterized by various cell types distributed within a three-dimensional ECM. Replicating this intricate architecture is challenging [6,36,37]. Tissue engineering through decellularization involves the removal of cellular antigens from the organ, preserving the extracellular matrix (ECM) to create a biological scaffold that maintains the native tissue architecture and vascular networks [38]. This method provides a biological structure that can potentially facilitate tissue regeneration and transplantation, as the preserved ECM mimics the natural environment of the pancreas [36,39]. In parallel, an essential aspect of pancreatic tissue engineering is the optimization of culture conditions to ensure the long-term survival and functionality of pancreatic cells [40]. This approach involves fine-tuning variables such as temperature, nutrient availability, and oxygenation, which are critical for sustaining the cells’ viability and secretory functions over time [41]. The advantages and disadvantages of each approach are summarized in Table 1.

Moreover, it is crucial to develop regulatory systems that replicate the feedback mechanisms of the human body to achieve precise glycemic control and prevent severe blood sugar fluctuations [40,42]. However, the main limitation in biofabricating an artificial endocrine pancreas lies in recreating the critical vascular flow of the organ, which makes in vivo transplantation particularly challenging [9,43,44]. Once transplanted, the pancreas must be safely and effectively integrated into the patient’s circulatory system. This involves tackling issues related to vascularization, mitigating immune response, and preventing complications such as blood clots or infections [41,43].

### 2.3. Traditional and Emerging Tissue Engineering Strategies Used to Build Bioartificial Organs, with an Emphasis on Specific Approaches Applied to the Pancreas

Well-established clinical methods, such as pancreatic transplantation, the infusion of islets of Langerhans, and more recently, the use of cellular drugs like Lantidra, have been central to therapeutic strategies aimed at restoring insulin production and improving glycemic control in patients whose β-cells have been destroyed by autoimmune processes in diabetes [45,46].

Among these methods, pancreas transplantation involves surgically implanting a donated organ, allowing the functional β-cells of the new pancreas to produce insulin [47]. This approach offers long-term glycemic control without the need for exogenous insulin. However, the procedure is associated with significant surgical risks and necessitates continuous immunosuppression to prevent organ rejection, which limits its widespread application [48].

In contrast, the infusion of Langerhans islets presents a less invasive alternative. This procedure transplants isolated pancreatic islets from cadaveric donors directly into the hepatic portal vein [49]. Although less invasive, it still requires long-term immunosuppression to prevent rejection of the islets. Studies indicate that many patients achieve insulin independence for several years, but the long-term success of this method is often hindered by the toxic effects of immunosuppressive drugs, which can compromise islet viability over time [50,51].

Building upon these methods, Lantidra^TM^ (Donislecel, Chicago, Il, USA) represents a novel cellular therapy recently approved by the FDA, designed for patients with difficult-to-control type 1 diabetes (DM1), particularly those experiencing severe hypoglycemia and difficulty regulating glucose levels [52,53]. Lantidra consists of isolated allogeneic pancreatic islets infused into these patients, offering the potential to reduce dependence on exogenous insulin and improve glycemic control. While initial clinical results are promising, as with other islet infusion therapies, long-term cell viability and the need for ongoing immunosuppression remain critical challenges that must be addressed to ensure sustained success [53,54].

**Figure 2:**
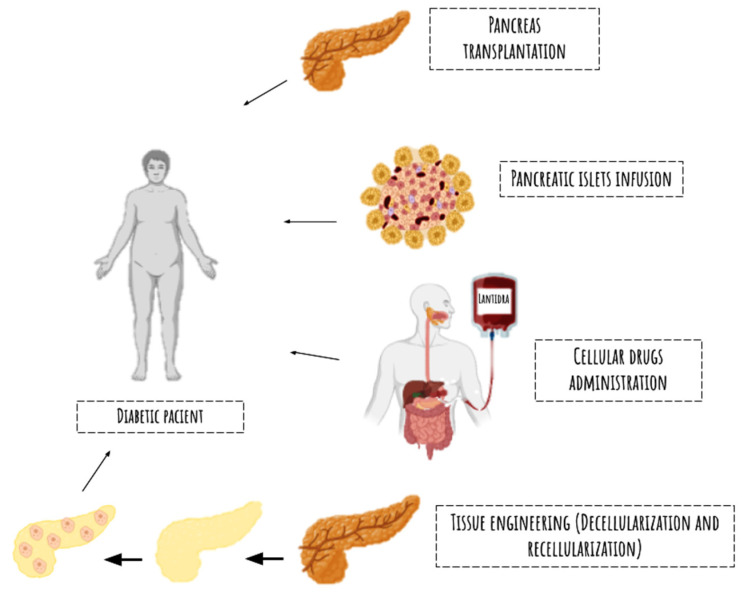
The image illustrates the various therapeutic methods aimed at restoring insulin production in diabetic patients, such as pancreatic transplantation, islet infusion, and the use of cellular drugs. In addition to tissue engineering, the decellularization process aims to address the shortage of organs for patients in need of transplants.

While these clinical methods continue to evolve, another promising avenue in the quest for solutions to organ failure is tissue engineering. Initially, this field for bioartificial organs began focusing on enhancing traditional organ transplantation techniques, such as those used for kidneys [55,56]. These approaches are summarized in Figure 2.

This technology was applied to pancreas and other organs, as the decellularization of whole organs preserves their three-dimensional architecture and allows recellularization with beta cell lines cultured over five days [57].

More recently, Chaimov et al. [14] proposed a new platform for insulin delivery tailored to diabetic patients. They developed a system for encapsulating an artificial pancreas using solubilized porcine pancreatic ECM. These ECM microcapsules were induced to differentiate into insulin-regulating cells, supporting their viability and differentiation and enhancing insulin delivery significantly.

Traditional strategies have evolved into emerging technologies for constructing bioartificial organs. Westenfelder et al. [58] demonstrated improved glycemic control intraperitoneally in diabetic mice by injecting new human pancreatic islets. Klak et al. [18] demonstrated the utility of 3D printing in organ construction, such as the pancreas, using a decellularized extracellular matrix in pigs, achieving high-quality outcomes.

## 3. Extracellular Matrix: Composition and Role in the Pancreas

The extracellular matrix provides essential structural and biochemical support, regulates molecular signaling, aids in tissue repair, and modulates inflammatory responses in organs like the pancreas [59,60,61,62,63]. Key basement membrane components in the pancreas include laminin, collagen IV, fibronectin, and entactin, which are critical for maintaining tissue integrity and supporting cellular adhesion, particularly in the islets of Langerhans [64,65]. Laminin plays a major role in β-cell attachment and signaling, which are vital for insulin production [59,64,65,66].

Pancreas-specific ECM components such as collagen types I, III, V, and glycosaminoglycans (GAGs) are crucial for structural support in both endocrine and exocrine tissues. Additionally, proteoglycans like perlecan regulate the diffusion of molecules that impact cell survival and function [65,67,68]. Recent studies in mice and pigs identified twelve different ECM proteins, including those from collagen types I, III, IV, V, and VI, and laminin β1 chains [59,69]. These findings are important for tissue regeneration and cancer research, particularly regarding fibronectin, which facilitates cell adhesion, migration, and differentiation, and is implicated in pancreatic tumor progression [70,71].

Growth factors, such as platelet-derived growth factor (PDGF) and transforming growth factor beta (TGF-β), play vital roles in tissue repair, with TGF-β being particularly involved in regulating cell proliferation and differentiation, and modulating the tumor microenvironment [72,73]. Integrins and selectins are the primary adhesion molecules involved, with integrins mediating ECM–cytoskeleton interactions, essential for cell attachment and response to ECM signals, and selectins (e.g., L-, P-, and E-selectin) facilitating leukocyte adhesion and immune responses [74,75,76,77,78].

### 3.1. The Critical Role of the ECM in Regulating Cell Differentiation, Migration, and Proliferation, as Well as in Maintaining Tissue Architecture

The ECM is a complex arrangement of molecules and macromolecules secreted by cells during tissue development. Once structured three-dimensionally, the ECM plays both structural and signaling roles [79]. The relationship between cells and the ECM is dynamic, as it directly influences stem cells and the formation of the tissue microenvironment [79,80]. Some studies have sought to understand this complexity and how the ECM controls the formation of the tissue microenvironment [20,80].

The physical properties of the ECM, such as rigidity and permeability, influence the migration and differentiation of stem cells. For mesenchymal cell differentiation, these influences are related to the availability of binding sites and the ability of integrins to recognize the physical properties of the ECM [81].

Pancreatic stem cells interact distinctly with the ECM. For pancreatic stem cell progenitors, the ECM guides migration and the formation of specific niches in the pancreas. In this microenvironment, they differentiate into more specialized progenitor cells, such as acinar cells or precursors of the islets of Langerhans, which house alpha (α) and beta (β) cells [82,83]. These islets are embedded within a dense network of ECM, which plays a critical role in cell function and survival. The ECM surrounding β-cells is rich in laminin and collagen IV, which support insulin secretion and β-cell differentiation. The ECM around α-cells is less studied, but it is thought to be influenced by similar ECM molecules, as both cell types coexist within the islets [84,85].

Laminin and fibronectin, for example, interact with membrane receptors on these cells, triggering signaling pathways that regulate their mobility and guide their migration to specific sites within the pancreatic tissue [86].

Mature pancreatic stem cells, like mesenchymal stem cells, are influenced differently by the ECM and are directed to sites of inflammation or injury in the pancreas. This migration is mediated by signaling through physical and chemical gradients in the ECM. Once in the target tissue, these cells can differentiate into functional cells, culminating in tissue repair [87]. Furthermore, the composition of the ECM can influence the differentiation of pancreatic mesenchymal stem cells into other lineages, such as exocrine pancreatic cells or insulin-producing beta cells. This results in functional changes during pancreatic regeneration and helps maintain organ homeostasis after tissue reconstitution [88,89].

### 3.2. Complex Interactions between Pancreatic Endocrine and Exocrine Cells and ECM Components, and How These Interactions Influence Normal and Pathological Pancreatic Function

Although many studies have focused on reconstructing the endocrine pancreas due to diabetes, less attention has been given to the reconstruction of the exocrine pancreas. This is particularly challenging in animal models, as the pancreatic excretory system varies substantially across species. Therefore, caution must be exercised when selecting an animal model that accurately replicates the characteristics of the target organ. In a previous study, we addressed the separation of the endocrine and exocrine pancreas and identified two distinct pancreatic portions among Xenarthra: one endocrine and one exocrine [90].

The ECM is a highly dynamic structure that undergoes constant remodeling. Its components are deposited, degraded, or modified [19], and abnormalities in this process contribute to deregulation of cell proliferation, invasion, death, and loss of differentiation, ultimately leading to fibrosis and cancer [91,92]. Understanding ECM remodeling mechanisms is fundamental to developing new strategies in tissue engineering and regenerative medicine.

A study investigated the cultivation of pancreatic islets with human pancreatic-derived ECM hydrogel. The results were promising, as the islets showed increases in glucose and KCl levels, stimulated insulin secretion, and improved mitochondrial function. The absence of ECM compromised these functions, thus underscoring the role of ECM in promoting the survival and physiology of human pancreatic islets [93].

In the pancreas, endocrine cells are grouped in the islets of Langerhans, which are dispersed among exocrine cells. Both cell types interact in various ways [6,11,94,95]. Exocrine cells produce digestive enzymes and secrete them into pancreatic ducts, which transport these enzymes to the duodenum for food digestion [95,96]. In contrast, endocrine cells produce hormones such as insulin and glucagon, which are released directly into the bloodstream to regulate blood glucose levels [94,97]. Both exocrine and endocrine cells share a common blood supply and are subject to hormonal and neural influences that can affect their function.

During development and in pathological conditions such as pancreatitis or pancreatic cancer, interactions between endocrine and exocrine cells can be disrupted. These alterations affect pancreatic homeostasis, leading to glandular dysfunction, tumor cell progression and invasion, and metastasis formation [98].

The pancreatic ECM plays a key role in the interactions between endocrine and exocrine cells, creating a three-dimensional microenvironment that affects their communication and function. It also modulates the availability of growth factors and cytokines, which can affect cellular mechanisms [99]. Proteins such as collagen, elastin, proteoglycans, and glycoproteins form the physical structure that supports cellular organization and facilitates communication through biochemical and mechanical signals [99].

## 4. Extracellular Matrix Engineering for Bioartificial Pancreas

The development of new biotechnologies, especially in cell cultivation and support, has significantly advanced therapeutic options for pancreatic comorbidities. Various strategies and techniques, such as the use of biomaterials, 3D printing, and tissue engineering, have been studied for several years to replicate the pancreatic ECM in bioartificial systems. These approaches involve the remodeling and degradation of ECM proteins by metalloproteinases MMP-2 and MMP-9 in models of induced pancreatitis [100].

Other techniques have also been used to investigate the pancreatic ECM and obtain a three-dimensional biological scaffold suitable for organ reconstruction (Table 2).

Other decellularization methods, using a combination of detergents, such as Triton-X100 and sodium dodecyl sulfate (SDS) by perfusion, have been employed in rats to obtain a decellularized ECM for 3D printing, while pigs were used to evaluated whole tissue decellularization followed by direct recellularization, respectively [37,102]. The main advantages and disadvantages of these techniques are summarized in Table 3.

The ECM and its proteins were characterized and quantified by Ma et al. [60]. These authors considered the matrix a regulatory element of the pancreatic microenvironment and compared fetal and adult matrix proteins. They also defined the role of these proteins in beta cell maturation.

According to Mantovani et al. [22], the production of pancreatic scaffolds must adhere to an ideal protocol and specific storage precautions, such as freezing at –80 °C and maintenance in PBSA at 4 °C, a phosphate–diphosphate saline supplemented with antibiotics and antifungals. Pancreatic islets soaked in hydrogel enhanced response to glucose and KCl, stimulating insulin secretion and improving their mitochondrial functions.

In addition, incorporating growth factors such as platelet-derived growth factor (PDGF) and transforming growth factor beta (TGF-β) may alternatively promote cell differentiation and pancreatic function [103]. The study of these cytokines is not new; Bottinger [104] evaluated the expression of the mutant-dominant negative TGF-β type II receptor in transgenic mice and found essential roles for TGF-beta in regulating growth and differentiation in the exocrine pancreas. Their study provided insights into the mechanisms by which the loss of responsiveness to TGF-β may promote the carcinogenic process, both directly through effects on cell proliferation and indirectly through positive regulation of associated TGF-β in a paracrine manner.

One of the most common approaches to conduct this procedure is by encapsulating growth factors in artificial ECM. Biofabrication techniques are employed during the manufacturing process to incorporate microspheres (or microgels), nanoparticles, and polymeric coatings containing the desired growth factors, which are gradually released to mimic the physiological microenvironment and promote cell differentiation [105,106].

Another possibility is to chemically modify the ECM to increase its affinity for specific growth factors, such as PDGF and TGF-β. Functional groups can be added to the matrix to enable specific binding and controlled release of these growth factors to the target cells, thus prolonging their biological activity [107,108].

Finally, an interesting approach is multicellular tissue engineering, in which target cells are pretreated with growth factors to increase their ability to differentiate into functional endocrine cells, such as insulin-producing beta cells [109,110]. However, further studies are needed to optimize and evaluate the safety and effectiveness of this approach in both preclinical and clinical models of pancreatic diseases, including diabetes mellitus.

## 5. Clinical Applications and Future Perspectives

Many studies have shown that a bioartificial pancreas incorporating pancreatic ECM offers promising treatments for type I diabetes [23,60,101,111]. Accordingly, several protocols have been developed to obtain functional scaffolds and even generate hydrogels [61,93]. Another area of research focuses on proteins such as biglycan, which act as markers for malignant cells capable of migrating to the digestive system, including the pancreas [111].

From a viability standpoint, these studies provide insights that support the idea that these structures can create favorable environments for cell support over extended periods while maintaining functionality [112,113,114]. Several studies have explored this capacity. For example, Lutz [25] discussed animal models for studying type 2 diabetes mellitus. Paolillo and Schinelli [115] addressed ECM changes during metastatic processes. Di Wu et al. [116] focused on constructing a microenvironment similar to the native pancreas, suitable for both cell growth and functional effort in decellularized murine scaffolds.

Despite these advances, researchers continue to face challenges primarily related to the safety and longevity of implants [117]. Immunological reactions, such as encapsulation around the implant, can compromise its long-term efficacy, prompting studies focused on enhancing the use of these devices [17].

Clinical studies to date have yielded valuable insights into the application of these technologies in a human context. Extensive exploration has been conducted on scaffolds for developing an artificial endocrine pancreas intended for transplantation into diabetic patients, with the results suggesting the feasibility of manufacturing an insulin-producing organ [112,118,119,120,121,122,123]. However, further research is needed to ensure the successful transplantation and functioning of this tissue-engineered organ as a secondary pancreas in both humans and animals. Some examples of studies that have explored strategies for developing a bioartificial pancreas are shown in Table 4.

Organic systems require careful consideration when introducing new components that can interact with their functional units, functioning harmoniously like new members of an orchestra. To this end, immunological interventions must be capable of minimizing the effects of the rejection of the bioartificial pancreas [3]. On the other hand, pancreatic islet transplantation requires a highly vascularized microenvironment. According to Nalbach et al. [6], the proteoglycan nerve/glial antigen 2 (NG2) expressed in pericytes is a crucial regulator of angiogenesis and increases the vascularization of pancreatic islets.

## 6. Concluding Remarks

Pancreatic bioengineering is emerging as a promising avenue in the search for innovative solutions to metabolic disorders such as diabetes mellitus. This article reviews the progress and obstacles faced by this discipline in its journey towards developing effective therapies. With artificial organs and tissue bioengineering continually improving, we are on the verge of a significant change in treating pancreatic diseases.

Artificial organs, such as encapsulated pancreatic islets, hold promise as less invasive and more effective treatments for diabetes patients. By protecting the transplanted cells from immune attack and allowing controlled insulin release, these devices offer considerable potential to improve patients’ quality of life. However, challenges such as biocompatibility and the longevity of the encapsulated cells must be overcome to ensure long-term effectiveness.

In future studies on pancreatic bioengineering using decellularized ECM, several challenges need to be addressed. One key issue is the immunogenicity of decellularized scaffolds, which can still trigger immune responses despite cell removal. Another critical challenge is ensuring sufficient vascularization to support long-term cell survival and function. Standardizing decellularization protocols is essential to preserve ECM components and maintain scaffold integrity. Additionally, selecting appropriate cells for recellularization and ensuring they replicate pancreatic functions effectively is a major focus. Lastly, ethical concerns and accessibility barriers, especially in low-resource settings, must be addressed to make these therapies widely available.

## Figures and Tables

**Table 1:** Comparison of tissue engineering methods for pancreatic regeneration.

Method	Advantages	Disadvantages
Tissue engineering through decellularization	Preserves the native structure of the pancreas; retains vascular networks, facilitating regeneration	Challenges in recellularizing the scaffold; potential residual immune response
Optimization of culture conditions for cell functionality	Greater control over culture conditions; can enhance cell longevity and functionality	Does not fully replicate the natural tissue environment; difficulty in maintaining optimal conditions over time

**Table 2:** Key studies investigating pancreatic ECM for the development of three-dimensional biological scaffolds.

Study Author and Year	Method	Main Results
Goh et al. (2013) [57]	Perfusion method to decellularize the entire pancreas, followed by recellularization with β-cells	Demonstrated insulin expression after recellularization, supporting potential for diabetes treatment
Theocharis et al. (2016) [20]	Detailed the structure of pancreatic ECM and its role in cell function	Highlighted ECM’s importance in cell survival, growth, and communication through adhesion receptors
Chaimov et al. (2017) [14]	Encapsulation of insulin-producing cells in porcine-derived ECM	Created a 3D fibrous niche that supports cell viability and differentiation for insulin delivery
Elebring et al. (2017) [101]	Cold perfusion of porcine pancreas, followed by human fetal pancreatic cell adherence	Achieved expression of endocrine (C-peptide, PDX1) and exocrine markers (glucagon, alpha amylase)
Wan et al. (2017) [10]	Use of induced pluripotent stem cells in a placental scaffold	Accelerated insulin expression compared to traditional plate cultures
Sackett et al. (2018) [61]	Production of hydrogel from decellularized human pancreas for cell culture	Developed a hydrogel for transplantation and cell culture, addressing vascular integration challenges
Xu et al. (2018) [13]	Incorporation of heparin in the decellularization process for reendothelialization	Enhanced scaffold stability and circulation of secreted factors, aiding in vascular regeneration

**Table 3:** Comparison between decellularized ECM for 3D printing and decellularized tissue followed by direct recellularization.

Technique	Advantages	Disadvantages
Decellularized ECM for 3D printing	Allows customization of tissue architecture; greater control over cell and ECM deposition, adapting the shape of the tissue	Creating a microenvironment as complex as that of natural tissue remains a challenge; it may require large quantities of decellularized ECM to create effective scaffolds
Decellularized tissue followed by direct recellularization	Maintains the native three-dimensional structure of the organ; provides greater support for cell adhesion due to the preservation of the ECM and key proteins such as collagen and elastin	It can be difficult to adequately recellularize the entire tissue; the process may be more time-consuming and requires advanced techniques for uniformity control

**Table 4:** The table shows some examples of a bioartificial pancreas, highlighting the methods and the results of its application.

Study Author and Year	Method	Main Results
Agudelo et al., 2009 [124]	Microencapsulation of islets in agarose hydrogel	The data indicated that cryopreserved Mic-islets transplanted as a bioartificial pancreas in diabetic mice restored normoglycemia and successfully controlled blood glucose levels for extended periods.
Yang et al., 2010 [125]	Mouse insulinoma cells encapsulating in agarose gel were enclosed in a calcium phosphate cement chamber to create a bioartificial pancreas (BAP)	The case report revealed that the BAPs implanted in the bone marrow cavity of a spontaneous diabetic feline were effective. The implanted BAPs provided therapeutic benefits despite sustained hyperglycemia.
Ludwig et al., 2012 [126]	Encapsulation of islet using a bioartificial microchamber	The work showed a minimally invasive implantable chamber normalized blood glucose in streptozotocin-induced diabetic rodents for up to 3 months. As a result of hypervascularization of the tissue surrounding the device, no relevant delay in insulin response to glucose changes has been observed.
Peloso et al., 2016 [31]	Decellularization with triton-based solution	The human pancreatic acellular extracellular matrix (hpaECM) scaffolds maintained the molecular and spatial template of the intact pancreas, were cytocompatible, and were able to modulate the immune response.
Salvatori et al., 2014 [119]	Decellularization using ionic and nonionic detergents, enzymatic nucleases and antimicrobials	The study outlines emergent technologies in regenerative medicine that may overcome the limitations of conventional diabetes therapies. Among them, novel decellularization protocols have allowed researchers to discover the advantages afforded by the native pancreatic extracellular matrix, proven to be an optimal platform for recellularization and whole-organ pancreas bioengineering to resolve the dire shortage of transplantable organs.
Ghosh et al., 2023 [127]	Three-dimensional (3D) bioprinting by extrusion	The review highlighted advances in islet encapsulation and 3D bioprinting for bioartificial pancreas development. Extrusion-based bioprinting (EBB) is noted for its versatility and ability to print high cell densities and various cell types. Coaxial extrusion bioprinting is particularly suited for pancreatic islet bioprinting, addressing immunoisolation and hypoxia through vascular network formation.
Li et al., 2023 [128]	Encapsulation of pancreatic islet in core–shell microgels and a pre-vascularized scaffold	As a result of the synergistic effect between anti-adhesive core–shell microgels and prevascularized hydrogel scaffold, the bioartificial pancreas can reverse the blood glucose levels of diabetic mice from hyperglycemia to normoglycemia for at least 90 days.
